# Danger Changes the Way the Mammalian Brain Stores Information About Innocuous Events: A Study of Sensory Preconditioning in Rats

**DOI:** 10.1523/ENEURO.0381-17.2017

**Published:** 2018-02-14

**Authors:** Nathan M. Holmes, Mukesh Raipuria, Omar A. Qureshi, Simon Killcross, Fred Westbrook

**Affiliations:** School of Psychology, University of New South Wales, Sydney, New South Wales 2052, Australia

**Keywords:** amygdala, associative learning, consolidation, danger, memory, sensory preconditioning

## Abstract

The amygdala is a critical substrate for learning about cues that signal danger. Less is known about its role in processing innocuous or background information. The present study addressed this question using a sensory preconditioning protocol in male rats. In each experiment, rats were exposed to pairings of two innocuous stimuli in stage 1, S2 and S1, and then to pairings of S1 and shock in stage 2. As a consequence of this training, control rats displayed defensive reactions (freezing) when tested with both S2 and S1. The freezing to S2 is a product of two associations formed in training: an S2-S1 association in stage 1 and an S1-shock association in stage 2. We examined the roles of two medial temporal lobe (MTL) structures in consolidation of the S2-S1 association: the perirhinal cortex (PRh) and basolateral complex of the amygdala (BLA). When the S2-S1 association formed in a safe context, its consolidation required neuronal activity in the PRh (but not BLA), including activation of AMPA receptors and MAPK signaling. In contrast, when the S2-S1 association formed in a dangerous context, or when the context was rendered dangerous immediately after the association had formed, its consolidation required neuronal activity in the BLA (but not PRh), including activation of AMPA receptors and MAPK signaling. These roles of the PRh and BLA show that danger changes the way the mammalian brain stores information about innocuous events. They are discussed with respect to danger-induced changes in stimulus processing.

## Significance Statement

How the brain stores innocuous information (e.g., the relationship between two neutral stimuli) is critically determined by (1) the presence of danger at the time of information processing, and (2) exposure to danger after information has been processed. In the absence of danger, storage (or consolidation) of innocuous information requires neuronal activity in the perirhinal cortex (PRh), but not the basolateral complex of the amygdala (BLA). In contrast, when the context is dangerous at the time of information processing, or became dangerous afterward, storage of the same information requires neuronal activity in the BLA, but not PRh. These results advance the field by providing the first demonstration that danger changes how innocuous information is consolidated in the medial temporal lobe (MTL).

## Introduction

The presence of danger elicits a suite of defensive reactions, including hyperarousal, attention toward cues that identify the danger, and preparation for “fight-or-flight.” Danger also biases our judgments, alters our perceptions and strengthens aspects of memory: e.g., traumatic experiences are confidently recalled and can be rich in detail (Brown and Kulik, 1977; Adolphs et al., 2005; McGaugh, 2006; Phelps, 2006; Kensinger, 2009). Neuroimaging studies in people suggest that the amygdala plays a central role in these various effects: e.g., danger increases activation of the amygdala (Buchel et al., 1998; Morris et al., 1999; Davis and Whalen, 2001; Whalen et al., 2004), and activity in this brain region correlates with memory for cues that signaled danger (LaBar et al., 1998; Sabatinelli et al., 2005; LaBar and Cabeza, 2006; Delgado et al., 2008). This suggestion has been confirmed in animal studies: they show that neuronal activity in the amygdala is necessary for the formation and consolidation of a cue-danger memory, as well as its subsequent retrieval, expression, and modification (e.g., reconsolidation; for reviews, see Schafe et al., 2001; Fanselow and Poulos, 2005; LeDoux, 2007; Johansen et al., 2011; Maren et al., 2013; Duvarci and Pare, 2014; Keifer et al., 2015).

In contrast to its role in coding information about danger, the amygdala is not typically required for coding information about neutral or innocuous events (Dwyer and Killcross, 2006; see also Balleine and Killcross, 2006). However, recent studies of sensory preconditioning in rats show that there are circumstances under which the amygdala is engaged for processing this type of information (Holmes et al., 2013). In these studies, rats were exposed to pairings of two innocuous stimuli, S2 and S1 (e.g., a sound followed by a light, counterbalanced), so-called sensory preconditioning. For some rats, S2-S1 pairings occurred in a familiar and safe context; for others, they occurred in a dangerous context, one in which they had been previously exposed to an innate source of danger (brief-but-aversive foot-shock). In each case, rats formed an S2-S1 association, as evidenced by the fact that subsequent training of S1 as a signal for shock resulted in freezing to that S1 as well as its associated S2 (for a description of the controls, see Holmes et al., 2013). However, the role of the amygdala in formation of the S2-S1 association depended on the nature of the context where the S2-S1 pairings occurred. Specifically, neuronal activity in the basolateral complex of the amygdala (BLA) was not required for encoding of the S2-S1 association in a familiar, safe context: under these circumstances, encoding of the S2-S1 association required activity (including NMDA receptor activation) in another region of the medial temporal lobe (MTL), the perirhinal cortex (PRh; Nicholson and Freeman, 2000). Conversely, neuronal activity in the PRh was not required for encoding of the S2-S1 association in a dangerous context: under these circumstances, encoding of the S2-S1 association required activity (including NMDA receptor activation) in the BLA.

In addition to showing that danger shifts processing of a sensory preconditioned association from the PRh to the BLA, our previous study showed that the shift is independent of the interval between danger and exposure to the new S2-S1 relation (e.g., it is equally evident when this interval is either 3 or 24 h), as well as the type of that relation (e.g., it is equally evident in acquisition or extinction of a sensory preconditioned association). However, as neuronal activity in the PRh or BLA was blocked before target training sessions, our previous study leaves open the question of whether the PRh and BLA play any role in consolidation of the S2-S1 association that forms in a safe or dangerous context, respectively; or alternatively, whether the PRh and BLA are exclusively involved in encoding the innocuous information in each case. Accordingly, the present study used the sensory preconditioning protocol described above to investigate how rats consolidate an innocuous S2-S1 association in the PRh and BLA. It addressed three major questions. The first was whether the MTL region that is critical for encoding the S2-S1 association is also critical for its consolidation. The second was whether consolidation of the associations formed in these brain regions shares synaptic and intracellular signaling requirements. The third was whether exposure to danger immediately after sensory preconditioning alters consolidation of the S2-S1 association in the PRh and/or BLA.

## Materials and Methods

### Subjects

Subjects were experimentally naïve, male, Sprague Dawley rats (390–510 g) obtained from a commercial supplier (Animal Resources Center). All experimental procedures were approved by the Animal Care and Ethics Committee at the University of New South Wales and in accordance with the National Institutes of Health Guidelines for the Care and Use of Laboratory Animals, revised 1996.

Rats were housed in plastic boxes (67 cm length × 40 cm width × 22 cm height) with food and water continuously available. There were four rats per home box. In each experiment, all groups were equally represented among rats in an individual home box. The boxes were located a climate-controlled colony room (lights on at 7:00 A.M.).

### Surgery and drug infusions

Before behavioral testing, rats were implanted with guide cannula directed toward the BLA or PRh. Rats were injected intraperitoneally with 1.3 ml/kg of the anesthetic ketamine (Ketapex; Apex Laboratories) at a concentration of 100 mg/ml and 0.3 ml/kg of the muscle relaxant xylazine (Rompun; Bayer) at a concentration of 20 mg/ml. Anesthetized rats were then mounted on a stereotaxic apparatus (David Kopf Instruments), and 26-gauge guide cannula (Plastics One) implanted through holes drilled in both hemispheres of the skull. The tips of the guide cannula were aimed bilaterally at one of two sites: BLA (anteroposterior: -2.4 mm; mediolateral: ±4.9 mm; dorsoventral: -8.2 mm); or PRh (anteroposterior: -4.15 mm; mediolateral: ±5.00 mm; dorsoventral: -8.3; angled at 9°; Paxinos and Watson, 1997). The guide cannulas were maintained in position with dental cement and dummy cannulas were kept in each guide at all times except during infusions. Immediately after the surgical procedure, rats were injected intraperitoneally with a prophylactic (0.4 ml) dose of 300 mg/kg solution of procaine penicillin. Rats were allowed 7 d to recover from surgery during which time they were handled and weighed daily.

Bupivacaine, 2,3-dihydroxy-6-nitro-7-sulphamoyl-benzo[f]quinoxaline-2,3-dione (NBQX), U0126, or vehicle was infused bilaterally in the BLA or PRh by inserting a 33 gauge internal cannula into the guide cannula. The internal cannula was connected to a 25-μl glass syringe attached to an infusion pump (Harvard Apparatus) and projected an additional 1 mm ventral to the tip of the guide cannula. A total volume of 0.5 μl was infused into either structure (BLA or PRh) at a rate of 0.25 μl/min. The internal cannula remained in place for an additional 2 min after the infusions and was then removed. One day before infusions, the dummy cannula was removed, and the infusion pump was turned on for 2 min to familiarize the rats with the procedure and thereby minimize stress on the infusion day.

The sodium channel blocker, bupivacaine hydrochloride (Sigma), and the AMPA receptor antagonist, NBQX (Sigma), were each dissolved in nonpyrogenic saline (0.9% w/v) to obtain final concentrations of 1 μg/μl. Nonpyrogenic saline (0.9% w/v) was used as a vehicle for experiments studying the effects of bupivacaine and NBQX. U0126 (Promega), a specific inhibitor of MAPK kinase (MEK), was dissolved in 100% dimethyl sulfoxide (DMSO; Sigma-Aldrich) to a final concentration of 4 µg/µl. MEK is an upstream regulator of ERK/MAPK activation (Favata et al., 1998). The stock was then diluted 1:1 in artificial CSF (ACSF; Tocris Bioscience). Vehicle (50% DMSO-ACSF) was prepared by diluting 100% DMSO 1:1 in ACSF.

### Histology

Subsequent to behavioral testing, rats received a lethal dose of sodium pentobarbital. The brains were removed and sectioned coronally at 40 μm through the BLA or PRh. Every second section was collected on a slide and stained with cresyl violet. The location of cannula tips was determined under a microscope by a trained observer, unaware of the subjects’ group designations, using the boundaries defined by the atlas of Paxinos and Watson (1997). Subjects with inaccurate cannula placements or with extensive damage were excluded from the statistical analysis.

### Apparatus

Each of the conditioning chambers used in this study measured 33 cm (height) × 31 cm (length) × 26 cm (width). The chambers were located in separate compartments of a wooden cabinet. The floor, walls and ceiling of the cabinet were black. The sidewalls and ceiling of the chambers were made of aluminum and the back and front walls were made of clear plastic. The floor consisted of stainless steel rods, 5 mm in diameter, spaced 10 mm apart, (center to center). A tray below the floor contained bedding material. A speaker mounted on the back wall of each cabinet was used for the presentation of a 1000-Hz pure tone at 75 dB (A scale) against a background noise of ∼45 dB measured by a digital sound level meter (Dick Smith Electronics, Australia). A set of LEDs was also mounted to the back wall of each cabinet and used for the presentation of a flashing light stimulus. A constant-current shock generator, which delivered unscrambled AC 50 Hz to the grid floor of the conditioning chamber, was used for the presentation of a moderate (0.5 mA, 0.5 s) or strong (0.8 mA, 0.5 s) foot-shock, as described below. An infrared light source illuminated each chamber (940 ± 25 nm) and a camera mounted on the back wall of each cabinet recorded the behavior of each rat. The camera was connected to a monitor and DVD recorder in another room of the laboratory. All stimulus presentations were controlled by appropriate software (MatLab, MathWorks Inc).

### Procedure

Rats were implanted with bilateral cannula targeting the PRh or BLA and allowed 7 d for recovery.

#### Context exposure

On each of days 1 and 2, rats received two sessions of context alone exposure (four sessions in total). Each session lasted for 20 min, and the two sessions on each day were separated by a minimum interval of 3 h. These sessions were intended to familiarize the rats with the context, and therefore, increase their attention to the auditory and visual cues presented in sensory preconditioning.

#### Additional context session

On day 3, rats were randomly allocated to one of five groups. Each group received two sessions of training. The first session lasted five min. During this time, two groups of rats were exposed to the context alone (groups safe-PRh and safe-BLA). Two groups were shocked twice in the context (groups danger-PRh and danger-BLA): each shock was delivered at 0.5 mA for 0.5 s, the first shock occurred 3 min after placement in the context and the second shock occurred 1 min later, i.e., 4 min after placement in the context. Among rats in the final group (group control), half were exposed to the context alone while the remainder were shocked in the context in the manner just described.

#### Sensory preconditioning

The second session of training on day 3 lasted 46 min. In this session, rats were exposed to eight paired presentations of two innocuous stimuli, S2 and S1. The tone and flashing light were counterbalanced across these stimulus identities, and within each group, equal numbers of rats received the tone or flash as S2 (and vice versa for S1). Presentations of S2 and S1 were such that offset of S2 coincided with onset of S1. Each presentation of S2 lasted for 30 s and each presentation of S1 lasted for 10 s. This was based on our previous research showing that these parameters are optimal for the formation of a sensory preconditioned association in stage 1 (Parkes and Westbrook, 2010; Holmes et al., 2013; Holmes and Westbrook, 2017). Onset of the first S2 presentation occurred 5 min after placement in the chamber, and the interval between each of the eight trials (defined from offset of S1 to onset of the next S2 presentation) was fixed at five min. After the last stimulus presentation (i.e., the final presentation of S1), rats remained in the context for a further 120 s before being returned to their home cages. Approximately 2 min later, they were taken to a separate room in the laboratory where they received a bilateral infusion of either bupivacaine (groups safe-PRh, safe-BLA, danger-PRh, or danger-BLA) or vehicle alone (group control). The details of these infusions are described above.

#### Fear conditioning of S1

On day 4, rats received two sessions of training in which they were exposed to two S1-shock pairings in each session, and the two sessions were separated by a minimum interval of 3 h. Each presentation of S1 lasted for 10 s and co-terminated in foot-shock, which was delivered at 0.8 mA for 0.5 s. In the first of the two sessions, onset of the first S1 presentation occurred 5 min after placement in the chamber. In the second session, onset of the first S1 presentation occurred 2 min after placement in the chamber. The interval to the second S1-shock pairing in each session was fixed at 11 min. Rats remained in the context for an additional 2 min after the final S1 presentation in each session.

#### Context extinction

On day 5, rats received two sessions of context extinction, which were intended to reduce the baseline level of context-elicited freezing. The details for these sessions were identical to those described for context alone exposure on days 1 and 2.

#### Testing

On day 6, rats received an additional 10-min session of context extinction to reduce spontaneous recovery of context-elicited freezing. Approximately 2 h later, rats were tested with eight presentations of S2 alone (i.e., under conditions of extinction). Each presentation of S2 lasted 30 s, the first S2 was presented 2 min after placement in the chamber and the interval between S2 presentations was fixed at three min. Rats remained in the context for an additional 2 min after the final S2 presentation. On day 7, rats were tested with eight presentations of the S1 alone (under extinction). Each presentation of the S1 lasted 10 s, the first S1 was presented 2 min after placement in the chamber and the interval between S1 presentations was fixed at three min. Again, all rats remained in the context for an additional 2 min after the final S1 presentation.

### Scoring and statistics

Freezing was defined as the absence of all movements except those related to breathing (Fanselow, 1980). Rats were observed every 2 s and scored as either freezing or not by two observers, one of whom was naïve to the purposes of the experiment. The correlation between the scores of the two observers was high, >0.9, and any discrepancies in the scores were resolved in favor of those by the naïve observer.

Freezing was scored across the first two minutes of each session to assess the baseline level of freezing to the context. It was additionally scored for the 30-s duration of each S2 presentation and the 10-s duration of each S1 presentation. The number of 2-s samples scored as freezing was expressed as a percentage of the total number of observations during the baseline, S2 and S1 periods. As there were no between-group differences in baseline levels of freezing in any of the experiments (*F* < 1.2), the data for each experiment are represented as difference scores (average level of freezing to S2 and S1 minus average level of freezing in the baseline) in each of the figures, and were analyzed using contrasts with repeated measures in ANOVA (Hays, 1963). The repeated measures were applied to training data, for which 'trials' was included as a variable. The test data were analyzed using the same contrast method but in the absence of any repeated measures (i.e., the data submitted to analysis was the mean levels of freezing across the eight test trials in each session). The criterion for rejection of the null hypothesis (α) was set at 0.05. Confidence intervals (95% for the mean difference, standardized using the sample SD) are also reported for each significant comparison in each experiment.

Across the set of experiments reported here, there were no significant differences in the test levels of freezing to S2 among control rats that had been exposed to S2-S1 pairings in either a safe or dangerous context [safe subgroup mean = 34.3% (SEM = 12.0); danger subgroup mean = 20.6% (SEM = 16.0), *F*s < 1], that had been shocked in the context either 10 min or 24 h after S2-S1 pairings [10 min subgroup mean = 25.4% (SEM = 13.0); 24 h subgroup mean = 21.7% (SEM = 9.1), *F*s < 1], or that had been infused with different vehicle solutions [NBQX vehicle subgroup means range from 33.0% to 46.9% (SEMs range from 4.9 to 6.7); U0126 vehicle subgroup means range from 25.4% to 28.7% (SEMs range from 6.1 to 11.3), *F*s < 2.1]. As a more powerful test of any such differences, we pooled across the vehicle-infused controls from each experiment to determine if the test levels of freezing to S2 differed among rats exposed to S2-S1 pairings in a safe context, a dangerous context, or a safe context that became dangerous. The mean percentage freezing to S2 was 35.1% (SEM = 5.8), 34.3% (SEM = 3.1), and 28.3% (SEM = 4.4%) in the safe, dangerous, and safe-dangerous subgroups, respectively. The differences between these subgroups were not statistically significant (largest *F* < 1.3). Hence, in each experiment, all vehicle-infused rats were combined to form a single composite control group.

Rats were excluded from each experiment if their cannulas were misplaced. The total numbers of exclusions in each experiment were, six in experiment 1, four in experiment 2A, 16 in experiment 2B, four in experiment 3A, 13 in experiment 3B, and five rats in experiment 4. The location of infusion cannula tips in each of the experiments is shown in Figure 5 (BLA on the left, PRh on the right).

## Results

### 

#### Experiment 1. The roles of the PRh and BLA in consolidation of an S2-S1 association depend on the status of the context at the time of sensory preconditioning

Experiment 1 examined whether danger regulates the neural substrates underlying consolidation of the S2-S1 association formed in sensory preconditioning. Briefly, half the rats were surgically prepared with cannulas targeting the PRh and the remaining rats were prepared with cannulas targeting the BLA. After recovery from surgery, all rats were then pre-exposed to the context (a distinctive chamber) on days 1 and 2, and then to pairings of S2 and S1 on day 3. The various groups in this experiment differed in their treatment before and after these S2-S1 pairings. Some rats received a shocked exposure to the context 3 h before the S2-S1 pairings, thus rendering the context dangerous when the pairings were experienced. Other rats received an equivalent period of context only exposure so that the context was merely familiar (and safe) at the time of sensory preconditioning. Finally, immediately after the S2-S1 pairings in the safe context, rats received an infusion of the sodium channel blocker, bupivacaine, into the PRh or BLA while other rats received an infusion of vehicle; likewise, immediately after the pairings in the dangerous context, rats received infusion of bupivacaine in the PRh or BLA while other rats received an infusion of vehicle. The infusions of bupivacaine were intended to silence neuronal activity in either the PRh or BLA (Hsu et al., 2002; Kleim et al., 2003; Stevenson, 2011; Leong and Packard, 2014).

All rats received pairings of S1 and shock on day 4, additional context exposure (to reduce baseline freezing) on day 5, and test presentations of S2 and S1 on days 6 and 7, respectively. The questions of interest concerned the levels of freezing to S2 and S1 among the various groups in these test sessions.

The baseline levels of freezing in the two test sessions were low (<15%), and did not significantly differ between the groups (all *F*s < 1). This shows that the context exposure sessions on days 1, 2, 5, and 6 of our protocol were highly effective in retarding conditioning to the context during its shocked exposures, and/or extinguishing any context-elicited freezing. This was the case for each of the experiments reported in this study (data not shown).

**Figure 1.**
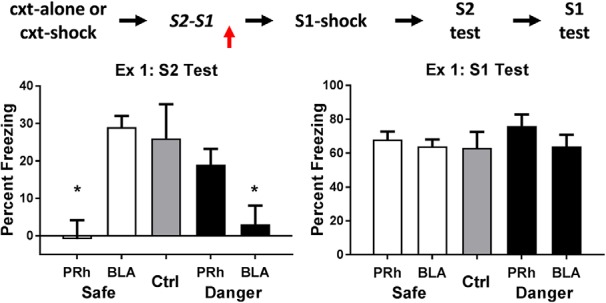
Experiment 1. The neural substrates involved in consolidation of a sensory preconditioned association depend on the context. The mean (±SEM) levels of freezing in groups control (*n* = 7), safe-BLA-bupi (*n* = 8), safe-PRh-bupi (*n* = 8), danger-BLA-bupi (*n* = 8), and danger-PRh-bupi (*n* = 8) during test presentations of S2 (left panel) and S1 (right panel) in experiment 1, relative to the baseline. When sensory preconditioning occurred in a safe context, consolidation of the S2-S1 memory required neuronal activity in the PRh but not the BLA; but when it occurred in a dangerous context, consolidation of the same memory required neuronal activity in the BLA but not the PRh. There was no evidence in any protocol that an infusion of bupivacaine impaired the encoding and/or storage of the S1-shock association. Horizontal arrows in the design schematic (top of figure) indicate transitions between experimental stages, and the vertical arrow indicates an infusion of bupivacaine or vehicle into the PRh or BLA.

The left and right panels of Figure 1 show the mean levels of freezing in each group during test presentations of S2 and S1, respectively. The levels of freezing among rats infused with saline were independent of their cannula placements and whether the sensory preconditioning (the S2-S1 pairings) occurred in a safe (spc-safe) or dangerous (spc-danger) context. Therefore, all rats infused with saline were combined to form a single composite control group (group Ctrl), which exhibited evidence for an S2-S1 memory (freezing to S2) and an S1-shock memory (freezing to S1). In contrast, the level of freezing among rats infused with bupivacaine depended on whether the context was safe or dangerous at the time of the S2-S1 pairings and on the brain region into which it was infused (*F*_(1,34)_ = 13.10, *p* < .05, 95% CI = [0.56, 2.00]). Specifically, when the S2-S1 pairings occurred in a safe context, consolidation of this association was impaired when bupivacaine was infused into the PRh, but was spared when bupivacaine was infused into the BLA: rats in group safe-PRh froze significantly less during test presentations of S2 than rats in groups safe-BLA and Ctrl (*F*_(1,34)_ = 13.18, *p* < .05, 95% CI = [0.7, 2.48]), and there was no difference in freezing between the latter groups (*F* < 1). In contrast, when the S2-S1 pairings occurred in a dangerous context, consolidation of this association was spared when bupivacaine was infused into the PRh, but was impaired when bupivacaine was infused into the BLA: rats in group danger-BLA froze significantly less during test presentations of S2 than rats in groups danger-PRh and Control (*F*_(1,34)_ = 6.15, *p* < .05, 95% CI = [0.2, 1.98]), and there was no difference in freezing between the latter groups (*F* < 1).

The doubly dissociable effects of the nature of the context (safe or dangerous) and the brain region inactivated by bupivacaine (PRh or BLA) show that the drug did not simply disrupt performance in the safe and dangerous protocols. Rats that received a bupivacaine infusion into the PRh or BLA were not impaired in their ability to freeze when tested with the sensory preconditioned S2. Moreover, the results of the test with the conditioned S1 (right panel) additionally show that any effects of PRh or BLA inactivation were selective to consolidation of the S2-S1 memory. Specifically, there were no differences in freezing to S1 among the groups that had been infused with bupivacaine (*F*s < 2.8), and the average level of freezing to S1 among these groups was similar to the freezing elicited by S1 in group control (*F* < 1).

These results show that the neural substrates underlying consolidation of an S2-S1 association depend critically on the nature of the context where this association was formed: in a familiar, safe context, consolidation requires neuronal activity in the PRh but not the BLA: in a dangerous context, consolidation requires activity in the BLA but not the PRh.

#### Experiment 2. Innocuous associations formed in the PRh and BLA share synaptic and intracellular signaling requirements

Experiment 2 further examined consolidation of the S2-S1 memory that forms in the safe and dangerous contexts and the substrates of this consolidation in the PRh and BLA, respectively. It specifically examined whether consolidation of the S2-S1 association that forms in these two distinct contexts requires neuronal processes that are necessary for consolidation of the association between S1 and shock (Johansen et al., 2011). It focused on two such processes: activation of AMPA receptors and activation of the MAPK signaling pathway. Both processes mediate changes in neurotransmission in cellular models of learning and memory (e.g., long-term potentiation), and within the BLA, consolidation of the memory that forms across pairings of S1 and shock (for AMPA receptor involvement, see Rumpel et al., 2005; Yeh et al., 2006; Nedelescu et al., 2010; Ota et al., 2010; Ganea et al., 2015; Lopez et al., 2015; for MAPK involvement, see Schafe et al., 2000; Ota et al., 2008; Ploski et al., 2008; Schafe et al., 2008; Monsey et al., 2011). Hence, we examined whether these processes also regulate consolidation of the association that forms across pairings of S2 and S1 by assessing whether blockade of these processes in the PRh and BLA disrupts consolidation of the S2-S1 association formed in the safe and dangerous contexts, respectively.

This experiment was conducted in two parts. Experiment 2A examined whether consolidation of the S2-S1 association formed in the safe context requires activation of AMPA receptors and MAPK signaling in the PRh. Three groups of rats were surgically prepared with cannulas targeting the PRh. After recovery from surgery, all rats were then pre-exposed to the context alone on days 1 and 2, and then exposed to pairings of S2 and S1 on day 3 (sensory preconditioning). The three groups differed in their treatment after these S2-S1 pairings. One group received an infusion of the AMPA receptor antagonist, NBQX; a second group received an infusion of U0126, an inhibitor of MEK1 and MEK2 which are upstream regulators of both extracellular signal-regulated kinases, ERK1 and ERK2; and the third group received an infusion of vehicle only (half the rats were infused with the vehicle for NBQX while the remaining rats were infused with the vehicle for U0126). All rats were exposed to S1-shock pairings on day 4, additional context exposure (to reduce baseline freezing) on day 5, and test presentations of S2 and S1 on days 6 and 7, respectively. The details for these training and test sessions were identical to those described in experiment 1 and our previous work (Holmes et al., 2013; Holmes and Westbrook, 2017).

**Figure 2.**
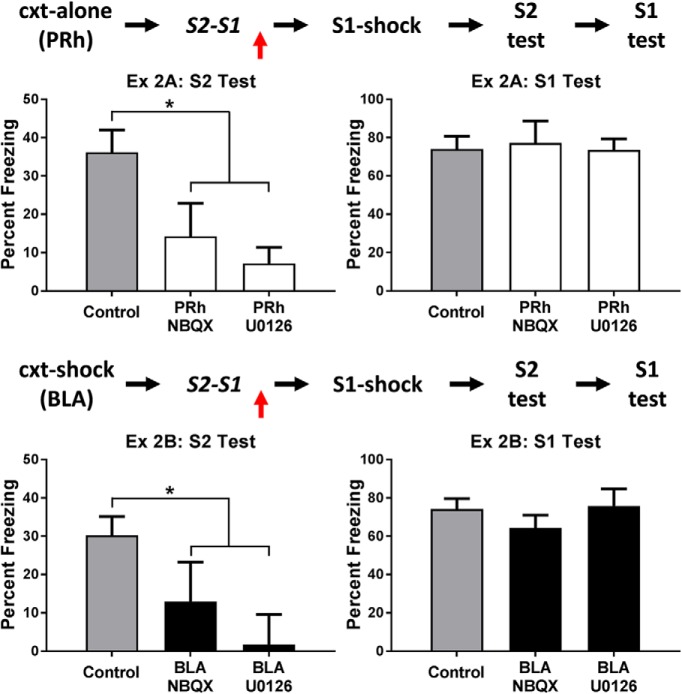
Experiment 2. The role of AMPA receptors and MAPK signaling in consolidation of an S2-S1 memory. The mean (±SEM) levels of freezing during test presentations of S2 (left panel) and S1 (right panel) in experiments 2A (top row) and 2B (bottom row), relative to the baseline. Experiment 2A: when sensory preconditioning occurred in a safe context, consolidation of the S2-S1 memory required activation of AMPA receptors and MAPK signaling in the PRh [groups vehicle (*n* = 12), NBQX (*n* = 8), and U0126 (*n* = 8)]. Experiment 2B: when sensory preconditioning occurred in a dangerous context, consolidation of the S2-S1 memory required activation of AMPA receptors and MAPK signaling in the BLA [groups vehicle (*n* = 15), NBQX (*n* = 8), and U0126 (*n* = 7)]. Horizontal arrows in the design schematics indicate transitions between experimental stages, and the vertical arrows indicate an infusion of NBQX, U0126 or vehicle into the PRh or BLA.

The top row of Figure 2 shows the mean levels of freezing in each group during test presentations of the sensory preconditioned S2 (left) and the conditioned S1 (right). It is clear that rats in the control group froze more during test presentations of S2 than rats in each of the drug treatment groups, but that all rats froze equally during test presentations of S1. The statistical analysis confirmed that rats in group control froze significantly more to S2 than rats in the two drug treatment groups (*F*_(1,25)_ = 11.56, *p* < .05, 95% CI = [0.51, 2.09]), and that there was no significant difference between the latter groups (*F* < 1). In the test of conditioning to S1, there was no significant difference in freezing between any of the groups (*F*s < 1). Thus, these results show that, when an S2-S1 association forms in a safe context, its consolidation requires activation of AMPA receptors and MAPK signaling in the PRh.

Experiment 2B examined whether consolidation of the S2-S1 association formed in a dangerous context requires activation of AMPA receptors and MAPK signaling in the BLA. Three groups of rats were surgically prepared with cannulas targeting the BLA. After recovery from surgery, all rats were pre-exposed to the context alone on days 1 and 2. On day 3, rats were shocked in the context (as described for groups danger-BLA and danger-PRh in experiment 1) and 3 h later returned to that context where they received S2-S1 pairings. The three groups differed in what was infused into the BLA after these S2-S1 pairings. One group received an infusion of the AMPA receptor antagonist, NBQX, a second group received an infusion of the MEK inhibitor, U0126, and the third group received an infusion of vehicle only (again, half the rats were infused with the vehicle for NBQX while the remaining rats were infused with the vehicle for U0126). Following S1-shock pairings on day 4, all rats were tested with S2 and S1 on days 5 and 6, respectively. The test results are shown in the bottom row of Figure 2. Rats in the control group again froze more to the sensory preconditioned S2 than rats in each of the drug treatment groups, but all rats froze equally during test presentations of the conditioned S1. The statistical analysis confirmed that rats in group control froze significantly more to S2 than rats in the two drug treatment groups (*F*_(1,27)_ = 7.68, p < .05, 95% CI = [0.26, 1.76]), and there was no significant difference in freezing between the latter groups (*F* < 1). There were no significant differences in freezing between any of the groups in the levels of freezing elicited by S1 (*F*s < 1.2). Thus, these results show that, when an S2-S1 association forms in a dangerous context, its consolidation requires activation of AMPA receptors and MAPK signaling in the BLA.

These results show that, although safe and dangerous environments recruit distinct brain regions to consolidate the S2-S1 association, activation of AMPA receptors and MAPK signaling are required in each of these regions for that consolidation: consolidation in a safe environment requires activation of AMPA receptors and MAPK signaling in the PRh; consolidation in a dangerous environment requires activation of AMPA receptors and MAPK signaling in the BLA.

#### Experiment 3. The experience of danger after sensory preconditioning shifts the substrates of consolidation from the PRh to the BLA

The previous experiments have shown that a dangerous context changes the brain regions that consolidate information about the innocuous S2 and S1. The present experiment examined two related questions: following exposure to S2-S1 pairings in a safe context, does the experience of danger release the PRh from its usual role in consolidation of the S2-S1 memory; and instead, does it engage the BLA for this consolidation?

This experiment was conducted in two parts. Experiment 3A examined whether the experience of danger immediately after S2-S1 pairings in a safe context affects consolidation of the S2-S1 memory in the PRh. Three groups of rats were surgically prepared with cannulas targeting the PRh. After recovery from surgery, all rats were then pre-exposed to the context alone on days 1 and 2, and then exposed to pairings of S2 and S1 on day 3 (sensory preconditioning). Exactly 10 min after the end of the sensory preconditioning session, all rats received an additional 5-min exposure to the context. The groups differed in their treatment during and after this brief context exposure. One group of rats was shocked twice in the context (in the manner described for groups danger in experiment 1), and then infused with bupivacaine into the PRh (group danger-Bupi). A second group of rats was exposed to the context but shock was not administered, and then infused with bupivacaine into the PRh (group safe-Bupi). Among the third group, half the rats were shocked in the context while the remainder were exposed to the context but were not shocked; both sets of rats were then infused with vehicle into the PRh (group control). On day 4, all rats were exposed to the context to extinguish any fear among the rats that had been shocked. All rats then received pairings of S1 and shock on day 5, context exposure (to reduce baseline freezing) on day 6, and test presentations of S2 and S1 on days 7 and 8, respectively. The details for these training and test sessions were identical to those described in experiment 1. We expected to replicate our previous finding that, in the absence of any other treatment, when an S2-S1 memory forms in a safe context, its consolidation requires neuronal activity in the PRh. This would be evidenced by more freezing to S2 in group control than in the group that received the context alone exposure after the S2-S1 pairings and a PRh infusion of bupivacaine. The question of interest concerned the level of freezing to S2 in the group that received a shocked exposure to the context after the S2-S1 pairings and a PRh infusion of bupivacaine. If the substrates involved in consolidation of the S2-S1 memory are determined at the time of the S2-S1 pairings, the experience of danger after these pairings should not alter the requirement for neuronal activity in the PRh. Therefore, the infusion of bupivacaine in these rats should disrupt consolidation of the S2-S1 association, evidenced by less freezing when tested with S2 in these rats than in control rats. If, however, the substrates involved in consolidation are influenced by the experience of danger after the S2-S1 pairings, then the infusion of bupivacaine into the PRh would fail to disrupt consolidation, evidenced by these rats freezing just as much as control rats when tested with S2.

**Figure 3.**
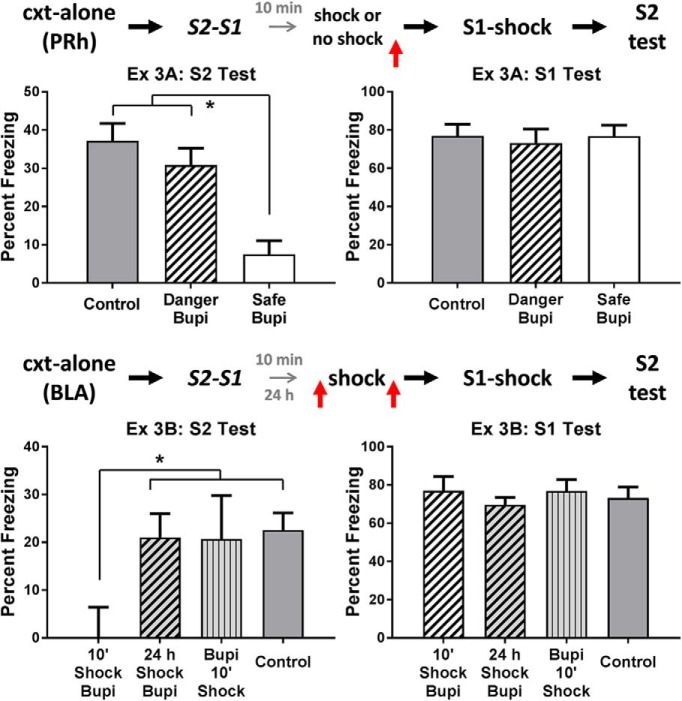
Experiment 3. Experience of danger shortly after a learning event changes how the brain stores information about that event. The mean (±SEM) levels of freezing during test presentations of S2 (left panel) and S1 (right panel) in experiments 3A (top row) and 3B (bottom row), relative to the baseline. Experiment 3A: a shocked exposure to the context after sensory preconditioning renders consolidation of the S2-S1 memory independent of neuronal activity in the PRh [groups vehicle (*n* = 8), danger-Bupi (*n* = 10), and safe-Bupi (*n* = 10)]. Experiment 3B: a shocked exposure to the context after sensory preconditioning renders consolidation of the S2-S1 memory dependent on neuronal activity in the BLA. This engagement of the BLA requires that the shocked context exposure occurs immediately after sensory preconditioning. Under these circumstances, the effect of the shocked context exposure is blocked when the BLA is inactivated before the shock [groups vehicle (*n* = 19), Bupi-shock-10 (*n* = 7) and shock-Bupi-10 (*n* = 7), and shock-Bupi-24 (*n* = 11)]. Horizontal arrows in the design schematics indicate transitions between experimental stages, and the vertical arrows indicate an infusion of bupivacaine or vehicle into the PRh or BLA.

The top row of Figure 3 shows the mean levels of freezing in each group during test presentations of S2 (left) and S1 (right) in experiment 3A. The statistical analysis of freezing elicited by the sensory preconditioned S2 confirmed what is clear from inspection of the left panel: rats infused with bupivacaine immediately after re-exposure to a familiar safe context froze significantly less than rats in the two other groups (*F*_(1,25)_ = 27.45, *p* < .05, 95% CI = [1.26, 2.89]). There was no significant difference between the latter groups (*F* < 1.2), that is, between the group infused with bupivacaine after the shocked exposure to what had been a safe context and the control group infused with vehicle. In the test of the conditioned S1, there were no significant differences in freezing between any of the groups (*F*s < 1). Thus, these results replicate our previous finding that neuronal activity in the PRh is critical for consolidation of an S2-S1 memory that forms in a safe environment. They additionally show that the experience of danger after S2-S1 pairings renders consolidation of the S2-S1 memory independent of neural activity in the PRh.

Experiment 3B examined three questions. The first was whether the experience of danger after S2-S1 pairings in a safe context engages the BLA in consolidation of the S2-S1 memory. The second was whether any effect of post-training danger in engaging the BLA for consolidation varies with the interval between the end of training and onset of the danger. The third question was whether any danger-induced recruitment of the BLA for consolidation of the S2-S1 memory can be blocked by inactivation of the BLA in advance of the danger. All rats were surgically prepared with cannulas targeting the BLA. After recovery from surgery, they were exposed to the context alone on days 1 and 2, and then to pairings of S2 and S1 on day 3. Following these pairings, rats were removed from the context and returned to their home cages. They were then returned to the context and shocked. The groups differed with respect to the interval between S2-S1 pairings and the shocked exposure to the context, and in their treatment before and after the shocked exposure to the context. One group received a shocked exposure to the context exactly 10 min after S2-S1 pairings (as per the details described for group danger in experiment 1), and this was immediately followed by a BLA infusion of bupivacaine (group 10’-Shock-Bupi). A second group received the same shocked exposure to the context exactly 24 h after S2-S1 pairings, and this was immediately followed by a BLA infusion of bupivacaine (group 24 h-shock-Bupi). A third group received the shocked exposure to the context exactly 10 min after S2-S1 pairings, and this was immediately preceded by a BLA infusion of bupivacaine (group Bupi-10’-shock). Among the fourth group, half the rats received a BLA infusion of saline immediately before the context-shock pairings while the remaining rats received this same infusion immediately after the context-shock pairings (group control). All rats were exposed to the context in the absence of shock to extinction any context-elicited fear responses on day 4 and to S1-shock pairings on day 5. All rats were again extinguished to the context on day 6, and tested with S2 and S1 on days 7 and 8, respectively.

The bottom row of Figure 3 shows the mean levels of freezing in each group during test presentations of S2 (left) and S1 (right) in experiment 3B. The statistical analysis confirmed what is clear from inspection of the figure. Rats infused with bupivacaine into the BLA 10 min after sensory preconditioning and the shocked exposure to the context (i.e., those in group 10’-shock-Bupi) froze significantly less when tested with the sensory preconditioned S2 than rats in the three other groups (*F*_1,40_ = 9.16, *p* < .05, 95% CI = [0.42, 2.11]). There were no significant differences between the levels of freezing elicited by S2 in control rats and in those that received the BLA bupivacaine infusions before the shocked exposure to the context, or that received the same infusion after a 24-h delayed session of context-shock pairings (*F* < 1). There were no significant differences among the groups in the levels of freezing elicited by the conditioned S1 (*F* < 1.5). These results indicate that the experience of danger after S2-S1 pairings shifted consolidation of the association from the PRh to the BLA, that the immediate, but not the delayed, experience of danger produces the shift, and that silencing the BLA before the experience of danger prevents this shift, allowing consolidation to occur in the PRh. With respect to the latter result, it suggests that silencing the BLA before the danger disrupts its processing in the BLA (i.e., it is as if the danger never occurred) and, therefore, recruitment of the BLA for consolidation of the S2-S1 association.

#### Experiment 4. The role of AMPA receptors and MAPK signaling in consolidation of the S2-S1 memory when danger occurs after the S2-S1 pairings

This experiment examined whether the danger-induced shift in consolidation requires activation of AMPA receptors and the MAPK signaling pathway within the BLA. All rats were surgically prepared with cannulas targeting the BLA. After recovery from surgery, they were then trained in the manner described for group 10’-shock-Bupi in experiment 3B. That is, rats were pre-exposed to the context on days 1 and 2, and then exposed to S2-S1 pairings on day 3. Shortly (10 min) after the S2-S1 pairings, all rats were returned to the context and shocked. The groups differed in their treatment after these context-shock pairings. One group received a BLA infusion of the AMPA receptor antagonist, NBQX. A second group received a BLA infusion of the MAPK antagonist, U0126. The third group received a BLA infusion of vehicle (half received the vehicle for NBQX while the remainder received the vehicle for U0126). All rats were exposed to sessions of context extinction on day 4, pairings of S1 and shock on day 5, context extinction on day 6, and test presentations of S2 and S1 on days 7 and 8, respectively.

**Figure 4.**
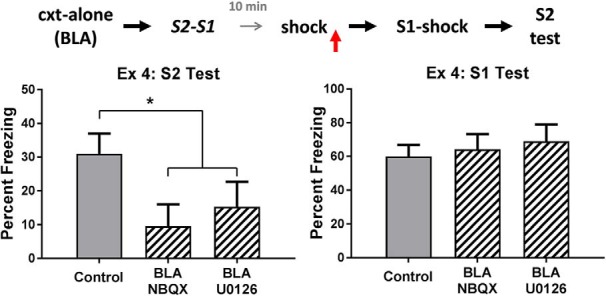
Experiment 4. The role of AMPA receptors and MAPK signaling in consolidation of an S2-S1 memory. The mean (±SEM) levels of freezing during test presentations of S2 (left panel) and S1 (right panel) in experiment 4, relative to the baseline. When danger is experienced shortly after sensory preconditioning, consolidation of the S2-S1 memory requires activation of AMPA receptors and MAPK signaling in the BLA. Groups vehicle (*n* = 15), NBQX (*n* = 8), and U0126 (*n* = 8). Horizontal arrows in the design schematic indicate transitions between experimental stages, and the vertical arrow indicates an infusion of NBQX, U0126 or vehicle into the BLA.

The top row of Figure 4 shows the mean levels of freezing in each group during test presentations of the sensory preconditioned S2 (left) and the conditioned S1 (right). It is clear that rats in the control group froze more during test presentations of S2 than rats in each of the drug treatment groups, and that there were no differences between the groups in their levels of freezing to S1. The statistical analysis confirmed that control rats froze more to S2 than rats treated with NBQX and U0126 (*F*_(1,28)_ = 5.75, p < .05, 95% CI = [0.13, 1.60]), and that there was no significant difference in freezing to S2 between the latter groups (*F* < 1). There were no significant differences among the groups in the test levels of freezing elicited by S1 (*F* < 1). These results show that, when danger occurs after S2-S1 pairings, activation of AMPA receptors and MAPK signaling in the BLA is required for consolidation of the S2-S1 memory, just as these processes are required for consolidation when danger occurs before the S2-S1 pairings (experiments 1 and 2B).

**Figure 5.**
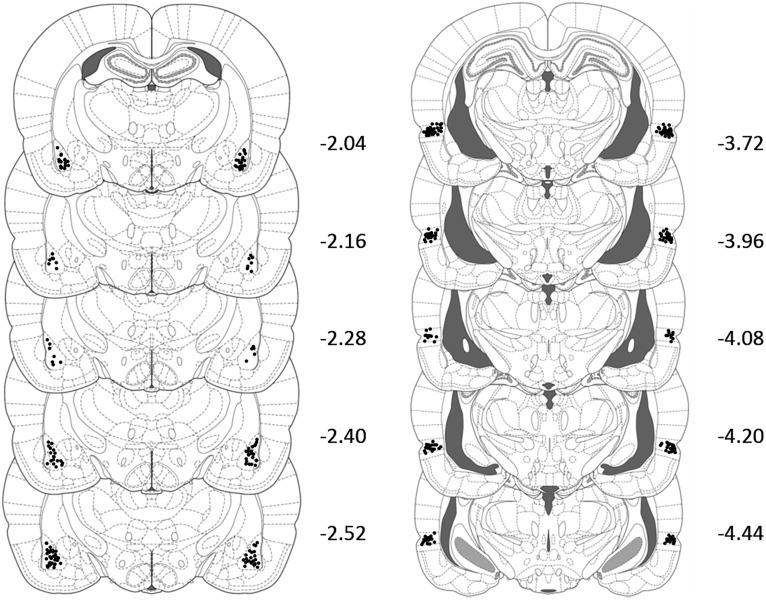
Approximate placements of microinfusion cannulas in the BLA for 124 rats (left) and PRh (right) for 76 rats. The cannula locations were verified on Nissl-stained coronal sections with reference to the atlas of Paxinos and Watson (1997; p 145).

### The effect of PRh and BLA drug infusions on context-conditioned freezing

In each of the experiments reported above, infusions of drugs into the PRh or BLA had variable effect on the levels of context-elicited freezing when rats were subsequently re-exposed to the context. PRh drug infusions had no effect on levels of context freezing in the next training session (*F*s < 1 in experiments 1 and 3A). BLA infusions of either U0126 or NBQX also spared the level of context-elicited freezing in the next training session: i.e., rats that received BLA infusions of either U0126 or NBQX after context-shock pairings (regardless of whether those pairing occurred before or after sensory preconditioning) froze just as much during the next training session as rats that received infusions of vehicle only (largest *F* = 3.3 in experiments 2B and 4). In contrast, rats that received BLA infusions of bupivacaine after context-shock pairings froze less than rats that received BLA infusions of vehicle only (smaller *F* = 5.41 in experiments 1 and 3B).

These findings suggest that neuronal activity in the BLA, but not PRh, is required for consolidation of fear to a highly familiar context (but see Wilensky et al., 2000; Huff et al., 2005) but that this requirement does not involve AMPA receptor-mediated signaling or ERK/MAPK signaling. However, the more important point for the present study is that the effect of BLA infusions of U0126, NBQX, and bupivacaine on consolidation of the S2-S1 association cannot be explained by appeal to their additional effects on consolidation of context conditioned freezing. That is, the disruptive effect of these infusions on consolidation of the S2-S1 association cannot be attributed to a disruption of the context fear memory, as the context fear memory was unaffected by the U0126 and NBQX infusions.

## Discussion

This series of experiments has shown for the first time that danger changes the way the brain consolidates information about innocuous events. The experiments used a sensory preconditioning protocol in which rats were first exposed to pairings of two innocuous stimuli, S2 and S1, and then to pairings of the S1 and shock (see also Parkes and Westbrook, 2010; Holmes et al., 2013). The rats showed defensive responses (freezing) when subsequently tested with the conditioned S1 and, more importantly, when tested with its associate, S2. We focused on the substrates of the S2-S1 memory in two brain regions within the MTL, the PRh, and BLA. We first showed that in a familiar, safe context, consolidation of the S2-S1 association requires neuronal activity in the PRh but not in the BLA. However, this was only true when the context was both familiar and safe. When the context was familiar but dangerous, rats learned the same S2-S1 association, but its consolidation required neuronal activity in the BLA but not in the PRh. The doubly dissociable roles of these brain regions in consolidation of the S2-S1 memory show that the drug used to probe their involvement, bupivacaine, did not simply alter the ability of the rats to freeze on the subsequent test. This conclusion was additionally supported by the absence of any drug effect on the formation of the S1-shock memory, regardless of the region into which it was infused. Instead, the results show that danger changes the way the brain stores innocuous information: it shifts the requirements for storage of this information from the PRh to the BLA.

The second major findings in this series of experiments is that danger also influences how the brain stores innocuous information that has already been encoded. Whereas consolidation of the S2-S1 memory formed in a safe context requires neuronal activity in the PRh and not the BLA, exposure to danger immediately after S2-S1 pairings spared memory formation but altered the requirements for its consolidation. Specifically, exposure to danger after the pairings removed the requirement for neuronal activity in the PRh and instead shifted the requirement to neuronal activity in the BLA. This shift was contingent on danger occurring shortly (a few minutes) after S2-S1 pairings; it did not occur when danger occurred 24 h after those pairings, and therefore, was not simply due to the history of context-shock pairings per se. Moreover, the shift was not due to any gross impairment in subsequent freezing by the drug, bupivacaine, used to silence the PRh or BLA. Instead, these results show that the experience of danger after the rats have encoded the S2-S1 association influences its consolidation in the same way as the presence of danger during the encoding of that association.

While the BLA is required for consolidation of an S2-S1 memory that forms in a context that is either already dangerous or that becomes dangerous immediately after S2-S1 pairings, the explanation for engagement of the BLA in each case is likely to be different. For example, the already dangerous context may alter the neural pathways by which information about S2 and S1 are transmitted through the brain, such that the point of their convergence shifts from the region where such information is processed in a safe context (the PRh) to other regions of the parahippocampal cortex (the entorhinal cortex; Paz et al., 2006; see also Albasser et al., 2010, 2011, 2015) and/or the BLA. This suggestion is supported by evidence from animal studies showing changes in early sensory processing of cues that predict danger (Kass et al., 2013). It is also supported by several lines of evidence from neuroimaging studies in people. Firstly, learned danger signals enhance activity in brain areas associated with early sensory processes (Sabatinelli et al., 2005; LaBar and Cabeza, 2006; Levita et al., 2015; Krusemark et al., 2013), as well as interactions between those areas and limbic regions associated with defensive reactions (Miskovic and Keil, 2012, 2013, 2014). Secondly, neural responses to danger vary with its proximity: as danger draws near, brain activity shifts from regions of the cortex (ventromedial prefrontal cortex) to regions of the limbic system (periaqueductal gray; Mobbs et al., 2007, 2009). Thirdly, and most importantly in light of the present findings, the context in which events occur determines the neural correlates of memory for those events: successful memory for events in a negative emotional context correlates with activation in the amygdala (Erk et al., 2003, 2005, 2010), whereas successful memory for events in a neutral context correlates with activation in parahippocampal regions of the cortex, including the PRh (Strange et al., 2002; Richardson et al., 2004). The results of experiments 1 and 2 extend these findings by showing that activation in the parahippocampal cortex and amygdala is necessary for consolidation of innocuous information in a safe and dangerous context respectively, and that within both of these brain regions, the cellular processes required for consolidation include activation of AMPA receptors and the MAPK signaling pathway.

However, in the case of the memory formed in a context that becomes dangerous after S2-S1 pairings, the shift in the substrates of its consolidation from the PRh to the BLA cannot be explained by a corresponding shift in the neural pathways by which S2 and S1 signals are transmitted through the brain. The context was safe when the association was formed, and hence, the association must have been processed in the same way up until the context was (or was not) rendered dangerous. Instead, the shift from the PRh to the BLA under these circumstances has two major implications. The first is that, even when the context is safe at the time of encoding, the newly formed S2-S1 association is represented in both the PRh and the BLA. This dual representation of the S2-S1 association may relate to the novelty of the two stimuli at the outset of sensory preconditioning: i.e., the novelty of S2 and S1 across the early trials of sensory preconditioning may dictate their processing within the BLA. However, as the stimuli are repeatedly experienced without consequence, their processing shifts from the BLA to the PRh. This suggestion is consistent with the well-established role of the amygdala in processing of novelty (Schwartz et al., 2003; Wright et al., 2003, 2008; Blackford et al., 2010; Balderston et al., 2011, 2013; Ousdal et al., 2014; Pedersen et al., 2017a,b), as well as the decline in amygdala activation as a novel stimulus becomes familiar across its presentations (Breiter et al., 1996; LaBar et al., 1998; Buchel et al., 1999; Morris et al., 2001; Phelps et al., 2004). Furthermore, a novel stimulus has been shown to activate different regions of the MTL as it becomes familiar (Strange et al., 1999, 2005a,b), and within the PRh, the increasing familiarity of a stimulus is reflected in distinct changes in the firing of PRh neurons (Kealy and Commins, 2011; Kent and Brown, 2012; Ranganath and Ritchey, 2012). The second major implication of these findings is that events that occur before or after S2-S1 pairings determine which of the PRh- and BLA-based representations is selected for consolidation. Specifically, our findings suggest that the PRh is usually selected for consolidation of the S2-S1 association; but that danger, either before or after formation of the S2-S1 association, biases selection of the BLA for consolidation (for similar arguments, see Okuda et al., 2004; Roozendaal et al., 2006). This selection of the BLA may alter the properties of the S2-S1 association that are stored and/or result in a stronger S2-S1 association (Roozendaal et al., 2006, 2008; Ritchey et al., 2017). Consistent with this claim, we have recently shown that what is learned in sensory preconditioning differs when it occurs in either a safe or dangerous context. Specifically, a dangerous context permits associative formation between spaced events in sensory preconditioning, and enhances discrimination between different events in sensory preconditioning (Holmes and Westbrook, 2017). It remains to be determined whether the experience of danger after sensory preconditioning reproduces these effects on sensory preconditioning in a dangerous context.

In summary, the present study has shown that danger changes the way the brain stores the S2-S1 memory that forms in sensory preconditioning: it shifts the neural substrates of this memory from the PRh, where it is otherwise stored, to the BLA. This was true when danger occurred before sensory preconditioning, and thus, the context was dangerous at the time of S2-S1 pairings; as well as when danger occurred after sensory preconditioning. However, in each case, the synaptic and intracellular processes involved in consolidation overlapped in terms of their requirements for AMPA receptor activation and MAPK signaling. These findings have two major implications. First, a dangerous context changes how S2 and S1 are trafficked through the brain, and therefore, their point of convergence in the MTL. Second, when a novel S2 and S1 are repeatedly paired in a safe context, some representation of their association exists within both the BLA and PRh, and which of these is selected for consolidation depends on whether danger is subsequently experienced or not.

## Synthesis

Reviewing Editor: Daniela Schiller, Mount Sinai School of Medicine Decisions are customarily a result of the Reviewing Editor and the peer reviewers coming together and discussing their recommendations until a consensus is reached. When revisions are invited, a fact-based synthesis statement explaining their decision and outlining what is needed to prepare a revision will be listed below. The following reviewer(s) agreed to reveal their identity: Denise Cai

The authors provided clarification in their rebuttal and can include some of those details in the methods section:

“To be perfectly clear, the data analysis involved contrasts with repeated measures in ANOVA. The repeated measures were applied to training data, for which ‘trials’ was included as a variable. The test data were analysed using the same contrast method but in the absence of any repeated measures (i.e., the data submitted to analysis was the mean levels of freezing across the eight test trials in each session).”

## Author Response

Dear Dr. Schiller,

Thanks very much for your consideration of our manuscript, titled “Danger changes the way the mammalian brain stores information about innocuous events: a study of sensory preconditioning in rats” (eN-TNWR-0381-17X).

We have addressed the point raised in the synthesis of reviews:

The authors provided clarification in their rebuttal and can include some of those details in the methods section: “To be perfectly clear, the data analysis involved contrasts with repeated measures in ANOVA. The repeated measures were applied to training data, for which ‘trials’ was included as a variable. The test data were analysed using the same contrast method but in the absence of any repeated measures (i.e., the data submitted to analysis was the mean levels of freezing across the eight test trials in each session).”

* This text now appears on page 11 of the revised manuscript (in blue text).

I hope that our manuscript is now suitable for publication in eNeuro.

Kind regards,

Nathan Holmes

## References

[B1] Adolphs R, Tranel D, Buchanan TW (2005) Amygdala damage impairs emotional memory for gist but not details of complex stimuli. Nat Neurosci 8:512–518. 10.1038/nn1413 15735643

[B2] Albasser MM, Poirier GL, Aggleton JP (2010) Qualitatively different modes of perirhinal-hippocampal engagement when rats explore novel vs. familiar objects as revealed by c-Fos imaging. Eur J Neurosci 31:134–147. 10.1111/j.1460-9568.2009.07042.x 20092559 PMC4235254

[B3] Albasser MM, Amin E, Iordanova MD, Brown MW, Pearce JM, Aggleton JP (2011) Perirhinal cortex lesions uncover subsidiary systems in the rat for the detection of novel and familiar objects. Eur J Neurosci 34:331–342. 10.1111/j.1460-9568.2011.07755.x 21707792 PMC3170480

[B4] Albasser MM, Olarte-Sánchez CM, Amin E, Brown MW, Kinnavane L, Aggleton JP (2015) Perirhinal cortex lesions in rats: novelty detection and sensitivity to interference. Behav Neurosci 129:227–243. 26030425 10.1037/bne0000049 PMC4450885

[B5] Balderston NL, Schultz DH, Helmstetter FJ (2011) The human amygdala plays a stimulus specific role in the detection of novelty. Neuroimage 55:1889–1898. 10.1016/j.neuroimage.2011.01.034 21256226 PMC3062695

[B6] Balderston NL, Schultz DH, Helmstetter FJ (2013) The effect of threat on novelty evoked amygdala responses. PLoS One 8:e63220. 10.1371/journal.pone.0063220 23658813 PMC3643910

[B7] Balleine BW, Killcross AS (2006) Parallel incentive processing: an integrated view of amygdala function. Trends Neurosci 29:272–279. 10.1016/j.tins.2006.03.002 16545468

[B8] Blackford JU, Buckholtz JW, Avery SN, Zald DH (2010) A unique role for the human amygdala in novelty detection. Neuroimage 50:1188–1193. 10.1016/j.neuroimage.2009.12.083 20045069 PMC2830341

[B9] Breiter HC, Etcoff NL, Whalen PJ, Kennedy WA, Rauch SL, Buckner RL, Strauss MM, Hyman SE, Rosen BR (1996) Response and habituation of the human amygdala during visual processing of facial expression. Neuron 17:875–887. 10.1016/S0896-6273(00)80219-6 8938120

[B10] Brown R, Kulik J (1977) Flashbulb memories. Cognition 5:73–99. 10.1016/0010-0277(77)90018-X

[B11] Buchel C, Morris J, Dolan RJ, Friston KJ (1998) Brain systems mediating aversive conditioning: an event-related fMRI study. Neuron 20:947–957. 9620699 10.1016/s0896-6273(00)80476-6

[B12] Buchel C, Dolan RJ, Armony JL, Friston KJ (1999) Amygdala-hippocampal involvement in human aversive trace conditioning revealed through event-related functional magnetic resonance imaging. J Neurosci 19:10869–10876. 10594068 10.1523/JNEUROSCI.19-24-10869.1999 PMC6784963

[B13] Davis M, Whalen PJ (2001) The amygdala: vigilance and emotion. Mol Psychiatry 6:13–34. 11244481 10.1038/sj.mp.4000812

[B14] Delgado MR, Nearing KI, Ledoux JE, Phelps EA (2008) Neural circuitry underlying the regulation of conditioned fear and its relation to extinction. Neuron 59:829–838. 10.1016/j.neuron.2008.06.029 18786365 PMC3061554

[B15] Duvarci S, Pare D (2014) Amygdala microcircuits controlling learned fear. Neuron 82:966–980. 10.1016/j.neuron.2014.04.042 24908482 PMC4103014

[B16] Dwyer DM, Killcross AS (2006) Lesions of the basolateral amygdala disrupt conditioning based on the retrieved representations of motivationally significant events. J Neurosci 26:8305–8309. 10.1523/JNEUROSCI.1647-06.2006 16899725 PMC6673804

[B17] Erk S, Kiefer M, Grothe J, Wunderlich AP, Spitzer M, Walter H (2003) Emotional context modulates subsequent memory effect. Neuroimage 18:439–447. 10.1016/S1053-8119(02)00015-0 12595197

[B18] Erk S, Martin S, Walter H (2005) Emotional context during encoding of neutral items modulates brain activation not only during encoding but also during recognition. Neuroimage 26:829–838. 10.1016/j.neuroimage.2005.02.045 15955493

[B19] Erk S, von Kalckreuth A, Walter H (2010) Neural long-term effects of emotion regulation on episodic memory processes. Neuropsychologia 48:989–996. 10.1016/j.neuropsychologia.2009.11.022 19945471

[B20] Fanselow MS (1980) Conditioned and unconditioned components of post-shock freezing. Pav J Biol Sci 15:177–182. 10.1007/BF03001163 7208128

[B21] Fanselow MS, Poulos AM (2005) The neuroscience of mammalian associative learning. Annu Rev Psychol 56:207–234. 10.1146/annurev.psych.56.091103.070213 15709934

[B22] Favata MF, Horiuchi KY, Manos EJ, Daulerio AJ, Stradley DA, Feeser WS, Van Dyk DE, Pitts WJ, Earl RA, Hobbs F, Copeland RA, Magolda RL, Scherle PA, Trzaskos JM (1998) Identification of a novel inhibitor of mitogen-activated protein kinase kinase. J Biol Chem 273:18623–18632. 10.1074/jbc.273.29.18623 9660836

[B23] Ganea DA, Dines M, Basu S, Lamprecht R (2015) The membrane proximal region of AMPA receptors in lateral amygdala is essential for fear memory formation. Neuropsychopharmacology 40:2727–2735. 10.1038/npp.2015.121 25915472 PMC4864648

[B24] Hays WL (1963) Statistics for psychologists. New York: Holt, Rinehart and Winston [WorldCat]

[B25] Holmes NM, Westbrook RF (2017) A dangerous context changes the way that rats learn about and discriminate between innocuous events in sensory preconditioning. Learn Mem 24:440–448. 28814470 10.1101/lm.044297.116 PMC5580528

[B26] Holmes NM, Parkes SL, Killcross AS, Westbrook RF (2013) The basolateral amygdala is critical for learning about neutral stimuli in the presence of danger, and the perirhinal cortex is critical in the absence of danger. J Neurosci 33:13112–13125. 10.1523/JNEUROSCI.1998-13.2013 23926265 PMC6619729

[B27] Hsu EH, Schroeder JP, Packard MG (2002) The amygdala mediates memory consolidation for an amphetamine conditioned place preference. Behav Brain Res 129:93–100. 10.1016/S0166-4328(01)00376-X 11809499

[B28] Huff NC, Wright-Hardesty KJ, Higgins E, Matus-Amat P, Rudy J (2005) Context pre-exposure obscures amygdala modulation of contextual fear conditioning. Lear Mem 12:456–460. 10.1101/lm.6705 16204200

[B29] Johansen JP, Cain CK, Ostroff LE, LeDoux JE (2011) Molecular mechanisms of fear learning and memory. Cell 147:509–524. 10.1016/j.cell.2011.10.009 22036561 PMC3215943

[B30] Kass MD, Rosenthal MC, Pottackal J, McGann JP (2013) Fear learning enhances neural responses to threat-predictive sensory stimuli. Science 342:1389–1392. 10.1126/science.1244916 24337299 PMC4011636

[B31] Kealy J, Commins S (2011) The rat perirhinal cortex: a review of anatomy, physiology, plasticity, and function. Prog Neurobiol 93:522–548. 10.1016/j.pneurobio.2011.03.002 21420466

[B32] Keifer OP, Hurt RC, Ressler KJ, Marvar PJ (2015) The physiology of fear: reconceptualizing the role of the central amygdala in fear learning. Physiology 30:389–401. 10.1152/physiol.00058.2014 26328883 PMC4556826

[B33] Kensinger EA (2009) Remembering the details: effects of emotion. Emot Rev 1:99–113. 10.1177/1754073908100432 19421427 PMC2676782

[B34] Kent BA, Brown TH (2012) Dual functions of perirhinal cortex in fear conditioning. Hippocampus 22:2068–2079. 10.1002/hipo.22058 22903623 PMC3445704

[B35] Kleim JA, Bruneau R, Calder K, Pocock D, VandenBerg PM, MacDonald E, Monfils MH, Sutherland RJ, Nader K (2003) Functional organization of adult motor cortex is dependent upon continued protein synthesis. Neuron 40:167–176. 10.1016/S0896-6273(03)00592-0 14527441

[B36] Krusemark EA, Novak LR, Gitelman DR, Li W (2013) When the sense of smell meets emotion: anxiety-state-dependent olfactory processing and neural circuitry adaptation. J Neurosci 33:15324–15332. 10.1523/JNEUROSCI.1835-13.2013 24068799 PMC3782615

[B37] LaBar KS, Cabeza R (2006) Cognitive neuroscience of emotional memory. Nat Rev Neurosci 7:54–64. 10.1038/nrn1825 16371950

[B38] LaBar KS, Gatenby JC, Gore JC, LeDoux JE, Phelps EA (1998) Human amygdala activation during conditioned fear acquisition and extinction: a mixed-trial fMRI study. Neuron 20:937–945. 10.1016/S0896-6273(00)80475-4 9620698

[B39] LeDoux J (2007) The amygdala. Curr Biol 17:R868–R874. 10.1016/j.cub.2007.08.005 17956742

[B40] Leong KC, Packard MG (2014) Exposure to predator odor influences the relative use of multiple memory systems: role of basolateral amygdala. Neurobiol Learn Mem 109:56–61. 10.1016/j.nlm.2013.11.015 24333118

[B41] Levita L, Howsley P, Jordan J, Johnston P (2015) Potentiation of the early visual response to learned danger signals in adults and adolescents. Soc Cogn Affect Neurosci 10:269–277. 10.1093/scan/nsu048 24652856 PMC4321623

[B42] Lopez J, Gamache K, Schneider R, Nader K (2015) Memory retrieval requires ongoing protein synthesis and NMDA receptor activity-mediated AMPA receptor trafficking. J Neurosci 35:2465–2475. 10.1523/JNEUROSCI.0735-14.2015 25673841 PMC6605616

[B43] Maren S, Phan KL, Liberzon I (2013) The contextual brain: implications for fear conditioning, extinction and psychopathology. Nat Rev Neurosci 14:417–428. 10.1038/nrn3492 23635870 PMC5072129

[B44] McGaugh JL (2006) Make mild moments memorable: add a little arousal. Trends Cogn Sci 10:345–347. 10.1016/j.tics.2006.06.001 16793325

[B45] Miskovic V, Keil A (2012) Acquired fears reflected in cortical sensory processing: a review of electrophysiological studies of human classical conditioning. Psychophysiology 49:1230–1241. 10.1111/j.1469-8986.2012.01398.x 22891639 PMC3422776

[B46] Miskovic V, Keil A (2013) Perceiving threat in the face of safety: excitation and inhibition of conditioned fear in human visual cortex. J Neurosci 33:72–78. 10.1523/JNEUROSCI.3692-12.2013 23283323 PMC3720233

[B47] Miskovic V, Keil A (2014) Escape from harm: linking affective vision and motor responses during active avoidance. Soc Cogn Affect Neurosci 9:1993–2000. 10.1093/scan/nsu013 24493849 PMC4249482

[B48] Mobbs D, Marchant JL, Hassabis D, Seymour B, Tan G, Gray M, Petrovic P, Dolan RJ, Frith CD (2009) From threat to fear: the neural organization of defensive fear systems in humans. J Neurosci 29:12236–12243. 10.1523/JNEUROSCI.2378-09.2009 19793982 PMC2782300

[B49] Mobbs D, Petrovic P, Marchant JL, Hassabis D, Weiskopf N, Seymour B, Dolan RJ, Frith CD (2007) When fear is near: threat imminence elicits prefrontal-periaqueductal gray shifts in humans. Science 317:1079–1083. 10.1126/science.1144298 17717184 PMC2648508

[B50] Monsey MS, Ota KT, Akingbade IF, Hong ES, Schafe GE (2011) Epigenetic alterations are critical for fear memory consolidation and synaptic plasticity in the lateral amygdala. PLoS One 6:e19958. [WorldCat] 21625500 10.1371/journal.pone.0019958 PMC3098856

[B51] Morris JS, Ohman A, Dolan RJ (1999) A subcortical pathway to the right amygdala mediating “unseen” fear. Proc Natl Acad Sci USA 96:1680–1685. 10.1073/pnas.96.4.1680 9990084 PMC15559

[B52] Morris JS, Buchel C, Dolan RJ (2001) Parallel neural responses in amygdala subregions and sensory cortex during implicit fear conditioning. Neuroimage 13:1044–1052. 10.1006/nimg.2000.0721 11352610

[B53] Nedelescu H, Kelso CM, Lázaro-Muñoz G, Purpura M, Cain CK, Ledoux JE, Aoki C (2010) Endogenous GluR1-containing AMPA receptors translocate to asymmetric synapses in the lateral amygdala during the early phase of fear memory formation: an electron microscopic immunocytochemical study. J Comp Neur 518:4723–4739. 10.1002/cne.22472 20963825 PMC3613289

[B54] Nicholson DA, Freeman JH (2000) Lesions of the perirhinal cortex impair sensory preconditioning in rats. Behav Brain Res 112:69–75. 10.1016/S0166-4328(00)00168-6 10862937

[B55] Okuda S, Roozendaal B, McGaugh JL (2004) Glucocorticoid effects on object recognition memory require training-associated emotional arousal. Proc Natl Acad Sci USA 101:853–858. 10.1073/pnas.0307803100 14711996 PMC321770

[B56] Ota KT, Pierre VJ, Ploski JE, Queen K, Schafe GE (2008) The NO-cGMP-PKG signaling pathway regulates synaptic plasticity and fear memory consolidation in the lateral amygdala via activation of ERK/MAP kinase. Learn Mem 15:792–805. 10.1101/lm.1114808 18832566 PMC2632793

[B57] Ota KT, Monsey MS, Wu MS, Schafe GE (2010) Synaptic plasticity and NO-cGMP-PKG signaling regulate pre- and postsynaptic alterations at rat lateral amygdala synapses following fear conditioning. PLoS One 5:e11236. 20574537 10.1371/journal.pone.0011236 PMC2888610

[B58] Ousdal OT, Andreassen OA, Server A, Jensen J (2014) Increased amygdala and visual cortex activity and functional connectivity towards stimulus novelty is associated with state anxiety. PLoS One 9:e96146. 10.1371/journal.pone.0096146 24755617 PMC3995962

[B59] Parkes SL, Westbrook RF (2010) The basolateral amygdala is critical for the acquisition and extinction of associations between a neutral stimulus and a learned danger signal but not between two neutral stimuli. J Neurosci 30:12608–12618. 10.1523/JNEUROSCI.2949-10.2010 20861367 PMC6633585

[B60] Paxinos G, Watson C (1997) The rat brain in stereotaxic coordinates. San Diego: Academic Press.

[B61] Paz R, Pelletier JG, Bauer EP, Paré D (2006) Emotional enhancement of memory via amygdala-driven facilitation of rhinal interactions. Nat Neurosci 9:1321–1329. 10.1038/nn1771 16964249

[B62] Pedersen WS, Balderston NL, Miskovich TA, Belleau EL, Helmstetter FJ, Larson CL (2017a) The effects of stimulus novelty and negativity on BOLD activity in the amygdala, hippocampus, and bed nucleus of the stria terminalis. Soc Cogn Affect Neurosci 12:748–757. 28008079 10.1093/scan/nsw178 PMC5460050

[B63] Pedersen WS, Muftuler LT, Larson CL (2017b) Disentangling the effects of novelty, valence and trait anxiety in the bed nucleus of the stria terminalis, amygdala and hippocampus with high resolution 7T fMRI. Neuroimage 156:293–301. 10.1016/j.neuroimage.2017.05.009 28502843 PMC5548630

[B64] Phelps EA (2006) Emotion and cognition: insights from studies of the human amygdala. Annu Rev Psychol 57:27–53. 10.1146/annurev.psych.56.091103.070234 16318588

[B65] Phelps EA, Delgado MR, Nearing KI, LeDoux JE (2004) Extinction learning in humans: role of the amygdala and vmPFC. Neuron 43:897–905. 10.1016/j.neuron.2004.08.042 15363399

[B66] Ploski JE, Pierre VJ, Smucny J, Park K, Monsey MS, Overeem KA, Schafe GE (2008) The activity-regulated cytoskeletal-associated protein (Arc/Arg3.1) is required for memory consolidation of pavlovian fear conditioning in the lateral amygdala. J Neurosci 28:12383–12395. 10.1523/JNEUROSCI.1662-08.2008 19020031 PMC6671728

[B67] Ranganath C, Ritchey M (2012) Two cortical systems for memory-guided behaviour. Nat Rev Neurosci 13:713–726. 10.1038/nrn3338 22992647

[B68] Richardson MP, Strange BA, Dolan RJ (2004) Encoding of emotional memories depends on amygdala and hippocampus and their interactions. Nat Neurosci 7:278–285. 10.1038/nn1190 14758364

[B69] Ritchey M, McCullough AM, Ranganath C, Yonelinas AP (2017) Stress as a mnemonic filter: interactions between medial temporal lobe encoding processes and post-encoding stress. Hippocampus 27:77–88. 10.1002/hipo.22674 27774683 PMC5436703

[B70] Roozendaal B, Okuda S, Van der Zee EA, McGaugh JL (2006) Glucocorticoid enhancement of memory requires arousal-induced noradrenergic activation in the basolateral amygdala. Proc Natl Acad Sci USA 103:6741–6746. 10.1073/pnas.0601874103 16611726 PMC1458951

[B71] Roozendaal B, Castello NA, Vedana G, Barsegyan A, McGaugh JL (2008) Noradrenergic activation of the basolateral amygdala modulates consolidation of object recognition memory. Neurobiol Learn Mem 90:576–579. 10.1016/j.nlm.2008.06.010 18657626 PMC2572617

[B72] Rumpel S, LeDoux J, Zador A, Malinow R (2005) Postsynaptic receptor trafficking underlying a form of associative learning. Science 308:83–88. 10.1126/science.1103944 15746389

[B73] Sabatinelli D, Bradley MM, Fitzsimmons JR, Lang PJ (2005) Parallel amygdala and inferotemporal activation reflect emotional intensity and fear relevance. Neuroimage 24:1265–1270. 10.1016/j.neuroimage.2004.12.015 15670706

[B74] Schafe GE, Atkins CM, Swank MW, Bauer EP, Sweatt JD, LeDoux JE (2000) Activation of ERK/MAP kinase in the amygdala is required for memory consolidation of pavlovian fear conditioning. J Neurosci 20:8177–8187. 11050141 10.1523/JNEUROSCI.20-21-08177.2000 PMC6772720

[B75] Schafe GE, Nader K, Blair HT, LeDoux JE (2001) Memory consolidation of Pavlovian fear conditioning: a cellular and molecular perspective. Trends Neurosci 24:540–546. 11506888 10.1016/s0166-2236(00)01969-x

[B76] Schafe GE, Swank MW, Rodrigues SM, Debiec J, Doyere V (2008) Phosphorylation of ERK/MAP kinase is required for long-term potentiation in anatomically restricted regions of the lateral amygdala in vivo. Learn Mem 15:55–62. 10.1101/lm.746808 18230673 PMC2216677

[B77] Schwartz CE, Wright CI, Shin LM, Kagan J, Whalen PJ, McMullin KG, Rauch SL (2003) Differential amygdalar response to novel versus newly familiar neutral faces: a functional MRI probe developed for studying inhibited temperament. Biol Psychiatry 53:854–862. 10.1016/S0006-3223(02)01906-6 12742672

[B78] Stevenson CW (2011) Role of amygdala-prefrontal cortex circuitry in regulating the expression of contextual fear memory. Neurobiol Learn Mem 96:315–323. 10.1016/j.nlm.2011.06.005 21689772

[B79] Strange BA, Fletcher PC, Henson RN, Friston KJ, Dolan RJ (1999) Segregating the functions of human hippocampus. Proc Natl Acad Sci USA 96:4034–4039. 10.1073/pnas.96.7.4034 10097158 PMC22415

[B80] Strange BA, Otten LJ, Josephs O, Rugg MD, Dolan RJ (2002) Dissociable human perirhinal, hippocampal, and parahippocampal roles during verbal encoding. J Neurosci 22:523–528. 11784798 10.1523/JNEUROSCI.22-02-00523.2002 PMC6758661

[B81] Strange BA, Duggins A, Penny W, Dolan RJ, Friston KJ (2005a) Information theory, novelty and hippocampal responses: unpredicted or unpredictable? Neural Netw 18:225–230. 10.1016/j.neunet.2004.12.004 15896570

[B82] Strange BA, Hurlemann R, Duggins A, Heinze HJ, Dolan RJ (2005b) Dissociating intentional learning from relative novelty responses in the medial temporal lobe. Neuroimage 25:51–62. 10.1016/j.neuroimage.2004.12.014 15734343

[B83] Whalen PJ, Kagan J, Cook RG, Davis FC, Kim H, Polis S, McLaren DG, Somerville LH, McLean AA, Maxwell JS, Johnstone T (2004) Human amygdala responsivity to masked fearful eye whites. Science 306:2061 10.1126/science.1103617 15604401

[B84] Wilensky A, Schafe G, LeDoux JE (2000) The amygdala modulates memory consolidaiton of fear motivated inhibitory avoidance learning but not classical fear conditioning. J Neurosci 20:7059–7066. 10995852 10.1523/JNEUROSCI.20-18-07059.2000 PMC6772812

[B85] Wright CI, Martis B, Schwartz CE, Shin LM, Fischer HH, McMullin K, Rauch SL (2003) Novelty responses and differential effects of order in the amygdala, substantia innominata, and inferior temporal cortex. Neuroimage 18:660–669. 10.1016/S1053-8119(02)00037-X 12667843

[B86] Wright CI, Negreira A, Gold AL, Britton JC, Williams D, Barrett LF (2008) Neural correlates of novelty and face-age effects in young and elderly adults. Neuroimage 42:956–968. 10.1016/j.neuroimage.2008.05.015 18586522 PMC2613685

[B87] Yeh SH, Mao SC, Lin HC, Gean PW (2006) Synaptic expression of glutamate receptor after encoding of fear memory in the rat amygdala. Mol Pharmacol 69:299–308. 16219906 10.1124/mol.105.017194

